# Dipteran (Bibionomorpha and Tipulomorpha) diversity in dead wood in Lithuania

**DOI:** 10.3897/BDJ.10.e85034

**Published:** 2022-10-11

**Authors:** Ina Gorban, Virginija Podeniene

**Affiliations:** 1 Life sciences center, Vilnius, Lithuania Life sciences center Vilnius Lithuania

**Keywords:** Diptera, Lithuania, dead wood, aspen, oak, ash, small-leaved lime, alder

## Abstract

The aim of this study is to compile the species list of Bibionomorpha and Tipulomorpha flies associated with dead wood in Lithuania. Saproxylic nematocerans were studied from 2014 to 2020 in four protected areas and in five different tree species *(Populustremula, Quercusrobur*, *Tiliacordata*, *Fraxinusexcelsior* and *Alnusglutinosa*) of the second stage of decay by using emergence traps. In total, 113 species were identified with Mycetophilidae, Sciaridae and Limoniidae being the most species-rich families. The compiled list of species emerging from dead wood in Lithuania is presented. Fourteen species were reared from dead wood for the first time.

## Introduction

Nematoceran flies – especially the species of infraorders Bibionomorpha (*sec.* Ševčík et al. 2016) and Tipulomorpha (*sec.* Wiegmann et al. 2011) – are a megadiverse group of insects and one of the most common groups associated with dead wood (Hövemeyer 1998). Infraorder Bibionomorpha consist of 17 extant families, most being mycetophagous or saprophagous (Ševčík et al. 2016). These families are the largest Diptera groups associated with fungal fruiting bodies and, in many cases, they are found in moist dead wood or under the bark of a trunk penetrated by fungal mycelia (Irmler et al. 1996, Hövemeyer 1998, Rotheray et al. 2001, Alexander 2002, Jakovlev 2011, Mlynarek et al. 2018, Ulyshen 2018). The infraorder Tipulomorpha is one of the largest groups in the suborder Nematocera, with five families Cylindrotomidae, Limoniidae, Pediciidae, Tipulidae and Trichoceridae (Wiegmann et al. 2011). They are found in various habitats, ranging from aquatic to terrestrial environments (Gelhaus and Podėnienė 2019). Some genera or species are obligatorily saproxylic, whereas for others, wood is just one possible habitat for their development (Krivosheina 1991, Hövemeyer and Schauermann 2003, Krivosheina 2006, Krivosheina and Zaitzev 2008, Podėnienė et al. 2012, Polevoi and Salmela 2014).

There is a large knowledge gap about the diversity of Bibionomorpha and Tipulomorpha in various tree species. Nematoceran diversity has, so far, been studied in only a few tree species, beech (*Fagussylvatica*) and aspen (*Populustremula*) being the most common (Hövemeyer 1998, Schiegg 2001, Hövemeyer and Schauermann 2003, Halme et al. 2012, Polevoi et al. 2018). The economic value of the aspen is very low; however, it holds a great diversity of saproxylic insects and is associated with rare species (Polevoi et al. 2018). Evidence related to the importance of the aspen for saproxylic insect diversity has also been provided by Finnish scientists (Halme et al. 2012). According to Irmler et al. (1996), in a study comparing beech, alder (*Alnusglutinosa*) and spruce (*Piceaabies*) dead wood, Sciaridae was most abundant in alder, while Mycetophilidae species were numerous in beech wood. A study of saproxylic Diptera in Scotland involving approximately 22 different tree species showed that birch (*Betulapubescens*), pine (*Pinussylvestris*) and aspen had the most diverse Diptera assemblage; however, only a few species of Tipulomorpha and none of Bibionomorpha were mentioned in the study (Rotheray et al. 2001). Study of dipterans in five tree species (*Fagussylvatica, Fraxinusexcelsior, Piceaabies, Populustremula* and *Quercusrobur*) by Økland (1999) showed microhabitats that are usually used by different species – logs, stumps, logs with different species of fungi etc. Although these studies present data on adults of many saproxylic species, many of these species still have unknown biology because it is unclear exactly where their larvae develop – this is especially true for those of the infraorder Bibionomorpha.

A decreased amount of dead wood in forest ecosystems because of forest clearance and habitat fragmentation can have a great impact on species, which can be put under threat. This paper compiles a list of nematoceran species in Lithuania reared from different tree species.

## Methods

**Figure 1.**
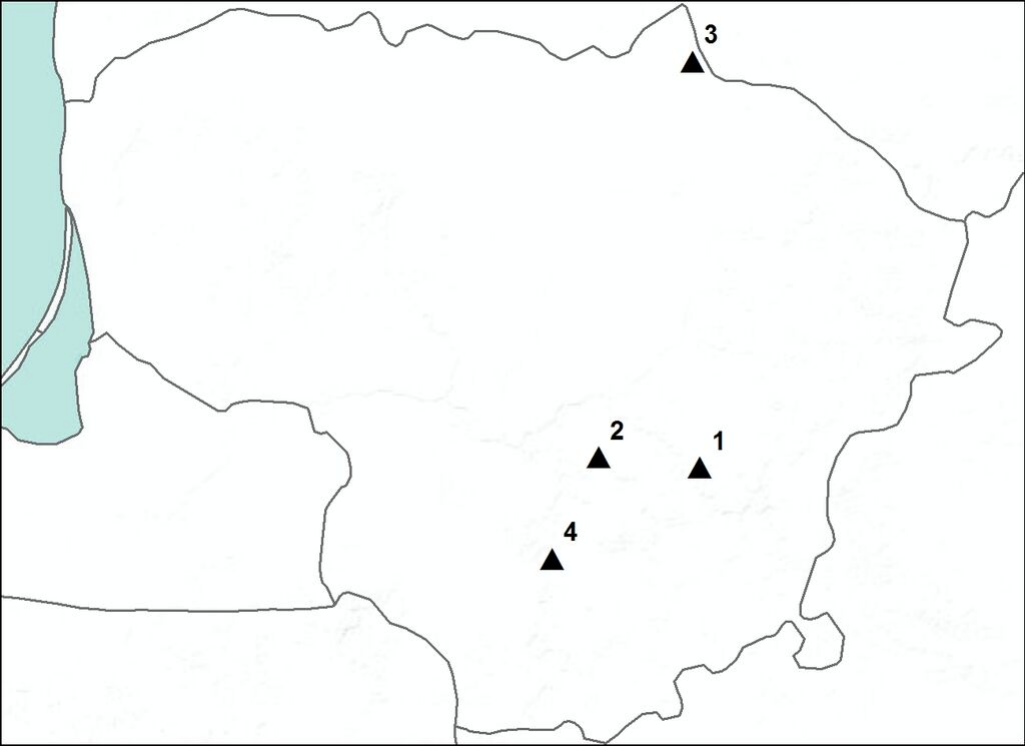
Nature Reserves in Lithuania. 1 - Dūkštų Ąžuolynas, Neries Regioninis Parkas Reserve, 2 – Būda Botanical-Zoological Reserve, 3 – Biržų Giria Botanical Reserve, 4 – Punia Šilas strict Nature Reserve.

The research was conducted in four nature reserves in Lithuania in 2014, 2016 and 2018 to 2020 (Fig. 1). In total, 40 traps were installed on tree trunks; however, six of them were empty. Five tree species were chosen – small-leaved lime (*Tiliacordata*), aspen (*Populustremula*), ash (*Fraxinusexcelsior*), alder (*Alnusglutinosa*) and oak (*Quercusrobur*) (Table 1). Saproxylic insects were reared using trunk-emergence traps (Gorban and Podėnienė 2021). Tent-like traps covered 1 m of fallen tree trunks, so a comparable section of every tree was used in the research. Traps were set in spring (April-May) and were kept until autumn (October-November); they were emptied every 10-14 days. As we do not know the exact time of tree death or fall, a classification table of wood decay was used. The wood of the second decay stage still has attached bark and fungal mycelia has penetrated 3 cm into the wood. We intentionally chose this decay stage because nematoceran larvae are common under the bark of the wood. Only males of the families Sciaridae and Mycetophilidae were included in the Table as the females are difficult to identify; the family Cecidomyiidae was also excluded.

## Results

In total, 808 specimens belonging to 113 species were identified (Table 2). The most abundant families were Sciaridae (204 specimens), Anisopodidae (179) and Mycetophilidae (150). The most species-rich families were Mycetophilidae (43 species), Sciaridae (26) and Limoniidae (22). Out of eight aspen trunks, 31 species and 338 specimens were collected; however, 158 specimens belonged to one species, *Sylvicolacinctus* (Anisopodidae). Out of seven oak trunks, 29 species and 99 specimens were reared, with Bibionidae being the most abundant (41 specimens). Out of 13 ash trunks, 54 species and 288 specimens were collected and Sciaridae was the most abundant (154 specimens). Out of three alder trunks, three species and four specimens were collected. Out of three linden tree trunks, 31 species and 79 specimens were collected, with Mycetophilidae being the most abundant and species-rich. In total, 17 species were reared from dead wood for the first time (Table 2, marked with an asterisk).

## Discussion

Our results show that communities of nematoceran flies in dead wood at the second stage of decay are species-rich and highly variable; however, more than half of the species were represented by a single specimen. Fourteen species were recorded from the dead wood for the first time.

Our study shows that dead wood is chosen by groups with a very different biology: obligate saproxylic species (*Gnophomyiaviridipennis*, Austrolimnophila (Austrolimnophila) ochracea, *Epiphragmaocellare*), mycetophagous species (Atypopththalmus (Atypophthalmus) inustus, *Achyrolimoniadecemmaculata*, *Discobolacaesarea, Metalimnobia* (*Metalimnobia*) *quadrinotata*, *M.quadrimaculata, Rhipidia* (*Rhipidia*) *maculata* and most species of the families Mycetophilidae, Ditomyiidae and Keroplatidae), species with a typical development habitat in moist soil or leaf litter (Dicranomyia (Glochina) tristis, *Limonianubeculosa*, *Limoniaphragmitidis*, Dicranophragma (Brachylimnophila) nemorale, *Ormosia (Ormosia) staegeriana, Rhypholophusbifurcatus*, *Rhypholophusvarius*, *Nephrotomaquadrifaria*, Tipula (Beringotipula) unca, *Trichoceraforcipula*, *Sylvicolacinctus* and most species of the families Bibionidae and Sciaridae) and species that develop under mosses (*Diogmaglabrata*, Tipula (Pterelachisus) apicispina, Tipula (Pterelachisus) luridorostris, Tipula (Pterelachisus) variicornis) (Irmler et al. 1996, Podėninė 2003, Nielsen and Nielsen 2007, Ševčík 2010, Jakovlev 2011, Jakovlev 2012, Seeber et al. 2012, Podėnienė 2012, Skartveit et al. 2013, Imada 2020). Once again, this shows the importance of dead wood not only for typical saproxylic insects, but also for other forest-dwelling species that choose wood only as a random site of development.

Previous studies of aspen (Halme et al. 2012, Polevoi et al. 2018, Gorban and Podėnienė 2021) showed a great variety of nematoceran species that depend on this tree species. In our study, *Sylvicolacinctus* (Anisopodidae) was the most abundant species in aspen, with some records from ash and oak wood. *S.cinctus* larvae is saprophagous and develops in decaying organic matter, also previously having been reared from aspen (Økland 1999). The second most abundant was *Gnophomyiaviridipennis* (Limoniidae). *Gnophomyia* is one of the first groups colonising dead trees and *G.viridipennis* specimens are repeatedly reared from aspen wood (Hancock 2008, Krivosheina 2008). *Dynatosomareciprocum* (Mycetophilidae) was reared mostly from aspen wood as well, with few specimens emerging from small-leaved lime trees. This species was previously reared from under the bark of spruce (Jakovlev 2011). *Ditomyiafasciata* (Ditomyiidae) specimens were reared from aspen trunks; however, it is common in various fungi species (Kurina and Ševčík 2006) and has previously been reared from alder wood (Irmler et al. 1996). *Zygoneurasciarina* (Sciaridae) and *Metalimnobiaquadrimaculata* (Limoniidae) also were reared only from aspen trunks. The larvae of *M.quadrimaculata* previously have been reared from decaying wood and from various fungi species (Ševčík 2003, Podėnienė 2012, Polevoi et al. 2018). Some species were reared only from small-leaved lime, for example, *Diogmaglabrata* (Cylindrotomidae), which is a well-known phytophagous species common in mosses in soil or dead wood (its larvae feed on mosses) and *Leiabimaculata* and Mycetophilaalea (Mycetophilidae), which are usually associated with different fungi species (Podėninė 2003, Krivosheina 2008, Kramer and Langlois 2019, Imada 2020). *Bibionigriventris* and *B.reticulatus* (Bibionidae) mostly were reared from oak and small-leaved lime. Adults of *B.reticulatus* are common in woodlands, where they usually occur in large numbers (Skartveit et al. 2013). Their larvae develop in strongly decomposed friable wood, decaying wood roots or forest litter (Krivosheina 2006). Tipula (Pterelachisus) apicispina specimens were mostly reared from oak as well. Usually, this species is found under moss cushions (Podėninė 2003).

Some species were reared only from ash wood. The most abundant species reared from ash wood was *Scatopsciaracalamophila* (Sciaridae), which previously had only been known from beech (Irmler et al. 1996, Schiegg 2001). Many specimens of Mycetophilidae family, also both Trichoceridae species (*Trichoceraforcipula* and *T.inexplorata*) were reared from ash trunks as well, although most of these species develop in fungi or decaying organic matter. The diversity in alder wood was the lowest and was represented only by four specimens and three species. Alder has been shown to have a much higher diversity in previous studies; for example, Irmler et al. (1996) reared many Mycetophilidae and Sciaridae from this tree species.

Although most of the species are known from different habitats, it appears that many of them use dead wood as one of their possible habitats, for example, *Exechiafusca* (Mycetophilidae) is known to develop in agarics (Jakovlev 1994) or soil and litter (Irmler et al. 1996); however, they also have been reared from strongly decayed pine wood (Jakovlev 2011). Decayed wood is penetrated by fungal mycelia and also can be covered with small patches of moss, providing suitable habitat for many species to develop. Since many species are represented by only one specimen, their preference for certain tree species needs to be studied further.

## Figures and Tables

**Table 1.** Emergence traps localities.

**Year**	**Nr. in the map**	**Reserve**	**Coordinates**	**Traps**	**Trap nr.**
**2014**	1	Dūkštų Ąžuolynas, Neries Regioninis Parkas Reserve	54°50'30.5"N 24°58'12.8"E	Aspen, Oak, Ash,	1 1 1
**2016**	2	Būda Botanical-Zoological Reserve	54°52'51.1"N 24°21'36.1"E	Ash, Aspen	4 1
**2018**	2	Būda Botanical-Zoological Reserve	54°52'51.1"N 24°21'36.1"E	Ash, Aspen	3 3
	3	Biržų Giria Botanical Reserve	56°15'03.6"N 24°57'40.4"E	Alder, Ash	2 2
**2019**	2	Būda Botanical-Zoological Reserve	54°52'51.1"N 24°21'36.1"E	Ash, Aspen	2 3
	3	Biržų Giria Botanical Reserve	56°15'03.6"N 24°57'40.4"E	Alder, Ash	1 1
**2020**	2	Būda Botanical-Zoological Reserve	54°52'51.1"N 24°21'36.1"E	Linden, Oak	1 3
	4	Punia Šilas strict Nature Reserve	54°31'48.8"N 24°04'50.4"E	Linden, Oak	2 3

**Table 2.** List of Bibionomorpha and Tipulomorpha species reared from different tree species (species reared from dead wood for the first time marked with asterisks).

	*Populustremula*	*Quercusrobur*	*Fraxinusexcelsior*	*Alnusglutinosa*	*Tiliacordata*
Number of traps	8	7	13	3	3
**Family Anisopodidae**					
*Sylvicolacinctus* (Fabricius, 1787)	158	1	20		
**Family Bibionidae**					
*Bibiomarci* (Linnaeus, 1758)		1			1
*Bibionigriventris* (Haliday, 1833)	1	28			11
*Bibioreticulatus* (Loew, 1846)		12	3		11
**Family Cylindrotomidae**					
*Diogmaglabrata (Meigen, 1818)					7
**Family Ditomyidae**					
*Ditomyiafasciata* (Meigen, 1818)	19				
*Symmerusannulatus* (Meigen, 1830)					2
*Symmerusnobilis* (Lackschewitz, 1937)			2		
**Family Keroplatidae**					
*Keroplatustestaceus* (Dalman, 1818)					1
*Neoplatyuraflava* (Macquart, 1826)			1		
*Orfeliafasciata* (Meigen, 1804)					2
*Orfelianemoralis* (Meigen, 1818)	1			2	1
**Family Limoniidae**					
*Achyrolimoniadecemmaculata* (Loew, 1873)					1
Atypopththalmus (Atypophthalmus) inustus (Meigen 1818)					1
Austrolimnophila (Austrolimnophila) ochracea (Meigen, 1804)					2
Dicranomyia (Dicranomyia) modesta (Meigen, 1818)			3		
*Dicranomyia (Glochina) tristis (Schummel,1829)					1
*Dicranophragma (Brachylimnophila) nemorale (Meigen, 1818)		1			
*Discobolacaesarea* (Osten Sacken, 1854)	1	1		1	
*Discobolaparvispinula* (Alexander, 1947)		2			
*Epiphragmaocellare* (Linnaeus, 1761)		1			
*Gnophomyiaviridipennis* (Gimmerthal, 1847)	72				
*Idiopterapulchella* (Meigen, 1830)			1		
*Limonianubeculosa* (Meigen, 1804)		1	1		
*Limoniaphragmatidis* (Schrank, 1781)					1
*Limoniatrivittata* (Schummel, 1829)			3		
*Metalimnobia* (Metalimnobia) *quadrimaculata* (Linnaeus, 1760)	11				
*Metalimnobia (Metalimnobia) quadrinotata (Meigen, 1818)					1
*Ormosia (Ormosia) staegeriana (Alexander, 1953)			1		
Rhipidia (Rhipidia) maculata (Meigen, 1818)			1		
*Rhypholophusbifurcatus (Goetghebuer, 1920)		2			
*Rhypholophusvarius* (Meigen, 1818)			2		
**Family Mycetophilidae**					
*Allodia (Brachycampta) grata (Meigen, 1830)					1
*Allodialugens* (Wiedemann, 1817)					1
*Allodiasubpistillata* (Ševčik, 1999)			1		
*Allodiatruncata (Edwards,1921)			1		
*Boletinacincticornis (Walker, 1848)		1			
Brachypeza (Brachypeza) armata (Winnertz, 1864)	1				
*Brevicornuserenum* (Winnertz, 1863)	1				
*Brevicornusericoma* (Meigen, 1830)					3
*Coelophthiniathoracica* (Winnertz, 1863)			1		
*Cordylabrevicornis* (Staeger, 1840)	1				
*Cordylapusilla* (Edwards, 1925)	1				
*Diadocidiaferruginosa* (Meigen, 1830)					1
*Dynatosomanigromaculatum* (Lundström, 1913)	2				
*Dynatosomareciprocum* (Walker, 1848)	26				2
*Exechiaconfinis* (Winnertz, 1863)			1		
*Exechiadizona* (Edwards, 1924)		1	7		
*Exechiadorsalis* (Staeger, 1840)		1	1		
*Exechiaexigua* (Lundstrom, 1909)			1		
*Exechiafusca* (Meigen. 1804)	1	1			6
*Exechianigroscutellata* (Landrock, 1912)			1		
*Exechiaunifasciata* (Lackschewitz, 1937)			3		
*Exechiaparva* (Lundström, 1909)		1	7		
*Exechiaparvula* (Zetterstedt, 1852)			22		
*Exechiaseriata* (Meigen, 1830)			2		
*Exechiarepandoides* (Caspers, 1984)			1		
*Exechiopsisfimbriata* (Lundstrom, 1909)			9		
*Gnoristebilineata* (Zetterstedt, 1852)			1		
*Leiabilineata* (Winnertz, 1863)			2		
*Leiabimaculata (Meigen, 1804)					4
*Leptomorphusforcipatus* (Landrock, 1918)	1				
*Mycetophilaalea* (Laffoon, 1965)					4
*Mycetophilafungorum* (De Geer, 1776)		1			2
*Mycetophilauliginosa* (Chandler, 1988)		1			
Mycomya (Mycomyopsis) permixta (Vaisanen, 1984)	1				
*Mycomyatenuis* (Walker, 1856)			2		1
*Notolophacristata* (Staeger, 1840)			1		
*Phroniabiarcuata* (Becker, 1909)					1
*Rymosiabifida* (Edwards, 1925)	2		9		
*Rymosiafasciata* (Meigen, 1804)		1	1		
*Rymosiaplacida* (Winnertz, 1863)			1		
*Saigusaiaflaviventris* (Strobl, 1894)	1				1
*Sciophilalimbatella* (Zetterstedt, 1852)		1			
*Sciophilalutea* (Macquart, 1826)	1				
**Family Sciaridae**					
*Bradysiafungicola* (Winnertz, 1867)		4			
*Bradysiapectoralis* (Staeger, 1840)			2		
*Bradysiaplacida* (Winnertz, 1867)		2			1
*Bradysiastrenua* (Winnertz, 1867)	1				
*Bradysiatrivittata (Staeger, 1840)		1			
*Corynopterabulgarica (Mohrig & Mamaev, 1992)		1			
*Corynopteradentata* (Bukowski and Lengersdorf, 1936)			5		
*Corynopteradeserta* (Heller and Menzel, 2006)			3		
*Corynopteraflavicauda* (Zetterstedt, 1855)					1
*Corynopteraforcipata* (Winnertz, 1867)			1		
*Corynopterafurcifera* (Mohrig and Mamaev, 1987)	1				
*Corynopterairmgardis* (Lengersdorf, 1930)			3		
*Corynopterapolana* (Rudzinski, 2009)			2		
*Corynopterasubtilis* (Lengersdorf, 1929)	3		8		
*Cratynanobilis* (Winnertz, 1867)	4		23		
*Epidapusdetriticola* (Kratochvil, 1936)	1	3	1		
*Epidapusgracilis* (Walker, 1848)		1			
*Epidapuslucifuga* (Mohrig, 1970)	1				
*Leptosciarellarejecta* (Winnertz, 1867)			1		
*Peyerimhoffiavagabunda* (Winnertz, 1867)					2
*Scatopsciaraatomaria* (Zetterstedt, 1851)	2		20		
*Scatopsciaracalamophila* (Frey, 1948)	3		70		
*Scatopsciarapusilla* (Meigen, 1818)			12		
*Xylosciaraheptacantha* (Tuomikoski, 1960)	1			1	
*Zygoneurabidens* (Mamaev, 1968)	9		2		
*Zygoneurasciarina* (Meigen, 1830)	7		1		
**Family Tipulidae**					
*Dictenidiabimaculata* (Linnaeus, 1760)		2			1
*Nephrotomaquadrifaria* (Meigen, 1804)			1		
Tipula (Pterelachisus) apicispina (Alexander, 1934)		21			4
Tipula (Platytipula) autumnalis (Loew, 1864)			12		
Tipula (Lunatipula) humilis (Staeger, 1840)			1		
Tipula (Pterelachisus) irrorata (Macquart, 1826)			2		
*Tipula (Pterelachisus) luridorostris (Schummel, 1833)		1			
*Tipula (Beringotipula) unca (Wiedemann, 1817)		4			
Tipula (Schummelia) variicornis (Schummel, 1833)	3				
Tipula (Pterelachisus) varipennis (Meigen, 1818)			1		
**Family Trichoceridae**					
*Trichoceraforcipula* (Nielsen, 1920)			3		
*Trichocerainexplorata* (Dahl, 1967)			1		
Total number of species	31	29	54	3	31
